# Evaluation of the Protective Efficacy of Foot-and-Mouth Disease Vaccines Against O/CATHAY Topotype Virus in Pigs

**DOI:** 10.3390/microorganisms14010186

**Published:** 2026-01-14

**Authors:** Ye-Ji Kim, Dong-Wan Kim, Mi-Kyeong Ko, Donghyeon Kim, Seo-Yong Lee, Yerin Kim, Yeonrea Chae, Tae-jun Kim, Hyejin Kim, Min Ja Lee, Sung-Han Park, Jaejo Kim, Jong-Hyeon Park, Ji-Hyeon Hwang, Yoon-Hee Lee

**Affiliations:** 1 Center for Foot and Mouth Disease Vaccine Research, Animal and Plant Quarantine Agency, 177 Hyeoksin 8, Gimcheon 39660, Republic of Korea; kimyeji7113@korea.kr (Y.-J.K.); dongwan12@korea.kr (D.-W.K.); mkk80@korea.kr (M.-K.K.); glass33@korea.kr (D.K.); happyhusband0406@korea.kr (S.-Y.L.); yerin3531@korea.kr (Y.K.); codusfo1@korea.kr (Y.C.); taejun0526@korea.kr (T.-j.K.); hyejin86@korea.kr (H.K.); herb12@korea.kr (M.J.L.); shpark1124@korea.kr (S.-H.P.); parkjhvet@korea.kr (J.-H.P.); 2 Research Planning Department, Animal and Plant Quarantine Agency, 177 Hyeoksin 8, Gimcheon 39660, Republic of Korea; jkim1209@korea.kr

**Keywords:** food-and-mouth disease, O/CATHAY topotype, vaccine, protective efficacy

## Abstract

The world is divided into seven regional pools based on the serotype distribution and geographical spread of the foot-and-mouth disease (FMD) virus. The Republic of Korea (ROK) belongs to Pool 1, where serotypes O, A, and Asia1 are endemic. Recently, the risk of incursions by the O/CATHAY topotype has increased in Pool 1, raising concerns about its potential introduction into the ROK. To assess the protective effectiveness of three commercial FMD vaccine strains—O1/Manisa + O/3039, O/Primorsky, and O1/Campos—currently used in the ROK against this topotype, an animal challenge experiment was conducted. Three treatment groups (*n* = 4 in each) of pigs received a single 2 mL injection of one of the vaccines at 8–10 weeks of age, and the other group (*n* = 2) served as the control. All pigs were challenged with the O/HKN/5/2019 virus (O/CATHAY topotype) at 21 days post-vaccination. All vaccines conferred protective effects, with O1/Campos demonstrating the highest efficacy by inducing fewest clinical signs and significantly reducing virus shedding in the treated groups compared with those in the control group. These findings suggest O1/Campos may serve as an emergency measure; nevertheless, the development of a vaccine specifically targeting the O/CATHAY topotype is warranted.

## 1. Introduction

Foot-and-mouth disease (FMD) is a highly contagious, severe animal disease in cloven-hoofed animals [1,2,3]. These animals include cattle, pigs, goats, and sheep [4,5]. Infected animals typically develop fever, lameness, and vesicular lesions on the feet and snout [6,7]. FMD negatively impacts productivity in adult animals and increases mortality among young animals, resulting in substantial economic losses [8,9,10]. FMD is caused by the foot-and-mouth disease virus (FMDV), classified under the family *Picornaviridae*, genus *Aphthovirus* [11,12]. FMDV is divided into seven serotypes: A, Asia1, C, O, SAT 1, SAT2, and SAT3 [13]. These seven serotypes are not uniformly distributed worldwide but instead predominate in specific regions or countries. Therefore, based on serotype and geographical distribution, the world is categorized into seven regional pools by the World Reference Laboratory for Foot-and-Mouth Disease (WRLFMD). The Republic of Korea (ROK) belongs to Pool 1 [14,15].

According to the Livestock Trend Survey conducted in the ROK in 2024, approximately 3.8 million heads of cattle were raised, of which approximately 90% comprised Korean native cattle and non-indigenous beef cattle, with the remaining 10% consisting of dairy cattle. For pigs, the population was estimated at around 10 million [16]. In endemic regions, vaccination programs are considered economically beneficial, with a benefit–cost ratio of 5.7, indicating that the economic losses due to FMD virus infection are nearly six times higher than the costs associated with vaccination [17].

To control FMD, the ROK has mandated FMD vaccination for cattle, pigs, and goats since 2011 [18,19]. All farms raising cattle, pigs, and goats receive government support covering 50% to 100% of vaccine purchase and vaccination costs, depending on their size and species. Vaccination with bivalent type O and A FMD vaccine is mandatory, and three commercial FMD vaccines are currently in use in the ROK. The antigens of the three commercial FMD vaccines are as follows: O1/Manisa + O/3039 + A22/Iraq, O/Primorsky + A/Zabaikalsky, and O1/Campos + A24/Cruzeiro + A/Argentina. Because each vaccine contains multiple antigens, this study focuses on the most relevant serotype O antigen for clarity and consistency.

According to the 2025 WRLFMD quarterly report [20], analysis of the relative prevalence of FMDV circulating in each Pool revealed that the prevalence of the O/CATHAY topotype in Pool 1 is increasing, indicating a heightened regional risk. In addition, O/CATHAY has been detected in the People’s Republic of China (PR China) and several Southeast Asian countries [21]. Given the geographic proximity of the ROK to PR China, the potential introduction of the O/CATHAY topotype into the ROK is of concern. Furthermore, vaccine-matching results reported in the 2019 WRLFMD quarterly report indicated that the O/CATHAY topotype exhibits a low antigenic matching value with some of the commercial vaccines currently used in the ROK [22]. Therefore, it is necessary to evaluate whether these vaccines provide sufficient protective efficacy in practice, which requires conducting animal challenge studies. In this study, we evaluated the protective effectiveness of three FMD vaccines (O1/Manisa + O/3039, O/Primorsky, and O1/Campos) against the O/CATHAY topotype (O/HKN/5/2019) in pigs.

## 2. Materials and Methods

### 2.1. Cells and Viruses

Porcine kidney (LF-BK) cells used in this study were obtained from the Plum Island Animal Disease Center (New York, NY, USA) [23]. The LF-BK cells were cultured in Dulbecco’s Modified Eagle Medium (Corning, Manassas, VA, USA) containing 10% fetal bovine serum (Atlas Biologicals, Fort Collins, CO, USA) and 1% Antibiotic–Antimycotic Solution (Corning, Manassas, VA, USA). Cells were grown in an incubator at 37 °C and 5% CO_2_.

The O/CATHAY/HKN/5/2019 (O/CATHAY) strain was used for the challenge infection and virus neutralization test (VNT). O/CATHAY/HKN/5/2019 was kindly provided by the Pirbright Institute (Woking, UK). The vaccine strains, O1/ME-SA/Manisa (O1/Manisa), O/CATHAY/3039 (O/3039), O/SEA/Primorsky (O/Primorsky), and O1/Euro-SA/Campos (O1/Campos) used for the VNT were generously provided by Boehringer Ingelheim (BI, Ingelheim, Germany), Federal Center for Animal Health (FGBI “ARRIAH,” Vladimir, Russia), and Servicio Nacional de Sanidad Animal (SENASA, Buenos Aires, Argentina).

### 2.2. Preparation of Commercial Vaccines

Three commercial vaccines with a potency exceeding 6 PD_50_ were obtained from the following manufacturers: O1/Manisa + O/3039 + A22/Iraq from Boehringer Ingelheim (BI, UK); O/Primorsky + A/Zabaikalsky from the Federal Center for Animal Health (FGBI “ARRIAH,” Russia); and O1/Campos + A24/Cruzeiro + A/Argentina from Biogenesis Bago (Garin, Argentina).

### 2.3. Animal Experiments

Animal experiments were performed within an animal biosafety level 3 (ABL3) facility at the Animal and Plant Quarantine Agency (IACUC approval number 2025-1612). An animal experiment consisting of four groups was performed to assess the protective effectiveness of three FMD vaccines against the O/CATHAY topotype. Three treatment groups (*n* = 4 in each) were vaccinated with a single dose (2 mL) of one of the three vaccines at 8—10 weeks of age. The control group (*n* = 2) was unvaccinated. Although a larger number of animals was initially desired, the group size was limited to four animals per group due to the capacity constraints of the ABL3 facility. All pigs were challenged with the O/HKN/5/2019 virus (O/CATHAY topotype) at 21 days post vaccination (dpv). Clinical symptoms were observed for 7 days following the challenge. The clinical score included a total of eight points, with one point assigned to each of the three hooves (excluding the inoculated hoof), the tongue, mouth, nose, lameness, and decreased appetite [24]. No separation was made for pigs exhibiting clinical symptoms, considering the field conditions. Serum samples were obtained every other day, while nasal and saliva swabs were collected daily from the day the challenge was initiated (Table 1).

### 2.4. Quantification of FMDV RNA in Serum, Nasal Swab, and Saliva Swab

Following the challenge, serum samples were collected every other day, whereas nasal and saliva swabs were collected daily. Viral RNA was extracted from these samples using the Maxwell RSC System (Promega, Madison, WI, USA). Real-time reverse transcription-polymerase chain reaction (rRT-PCR) was carried out using the AccuPower FMDV Real-Time RT-PCR MasterMix Kit (Bioneer, Daejeon, Republic of Korea) and CFX96™ Real-Time System (Bio-Rad, Hercules, CA, USA). The rRT-PCR reactions were performed in a total reaction volume of 50 µL, consisting of 44 µL FMDV MasterMix, 1 µL internal positive control, and 5 µL extracted RNA [25]. Reverse transcription was performed at 45 °C for 30 min, followed by an initial denaturation at 95 °C for 5 min and 45 cycles of denaturation at 95 °C for 15 s and annealing/elongation at 55 °C for 50 s. Fluorescence from 6-carboxyfluorescein-labeled probes was measured at the end of each annealing/elongation step.

### 2.5. Enzyme-Linked Immunosorbent Assay (ELISA)

The VDPro FMDV SP O ELISA Kit (Median Diagnostics, Chuncheon, Republic of Korea) was used to identify antibodies targeting the structural protein (SP) of FMDV in pigs. This in vitro diagnostic assay is a blocking ELISA based on monoclonal antibodies reacting with recombinant antigens of FMDV serotype O, including the VP1 protein. The assay enables the detection of antibodies induced by vaccination or infection with FMDV serotype O. All ELISA procedures were performed following the manufacturer’s instructions, and all samples were tested in duplicate. Optical density (OD) values were measured at 450 nm and converted to S/N values using the following formula:

(1) $$S/N=\frac{\left(OD\;of\;sample-Mean\;OD\;of\;positive\;control\right)}{\left(Mean\;OD\;of\;negative\;control-Mean\;OD\;of\;positive\;control\right)}$$

For ease of interpretation, the values were transformed to 1 − S/N. Samples with 1 − S/N values ≥ 0.4 were interpreted as positive for FMDV type O SP antibodies, whereas those with values < 0.4 were considered negative.

### 2.6. VNT

VNT was conducted in accordance with the World Organization for Animal Health (WOAH) protocol (2022, CHAPTER 3.1.8). VNTs were performed against both the challenge virus (O/HKN/5/2019) and the vaccine strain viruses to assess changes in neutralization antibody titers. The following viruses were used: O/HKN/5/2019, O1/Manisa, O/3039, O/Primorsky, and O1/Campos. Serum samples were heat-inactivated at 56 °C for 30 min, followed by preparation of serial dilutions in microplates. Each VNT was conducted in duplicate. Virus suspensions containing 200 TCID_50_ (50% tissue culture infectious dose) were added to each well and incubated for 1 h. Subsequently, LF-BK cells were seeded into each well and incubated for 3 days. Equal volumes (50 µL) of diluted serum, virus, and LF-BK cells were used. Neutralizing antibody titers were calculated using the Spearman–Kärber method and expressed as log_10_ values [24,26]. Titers ≥ 1.2 were considered positive for neutralizing antibodies [27].

### 2.7. Statistical Analysis

Clinical scores, S/N values, and virus neutralization (VN) titers were analyzed using GraphPad Prism version 9.5.0 (GraphPad Software, CA, USA) and are presented graphically. All data are expressed as the mean ± standard error of the mean. A mixed-effects analysis was used for ELISA data, and a two-way repeated measures analysis of variance was applied for VNT data. Tukey’s multiple-comparisons test was performed for post hoc analyses.

## 3. Results

### 3.1. Clinical Signs and Virus Shedding Following Challenge Infection

**Figure 1:**
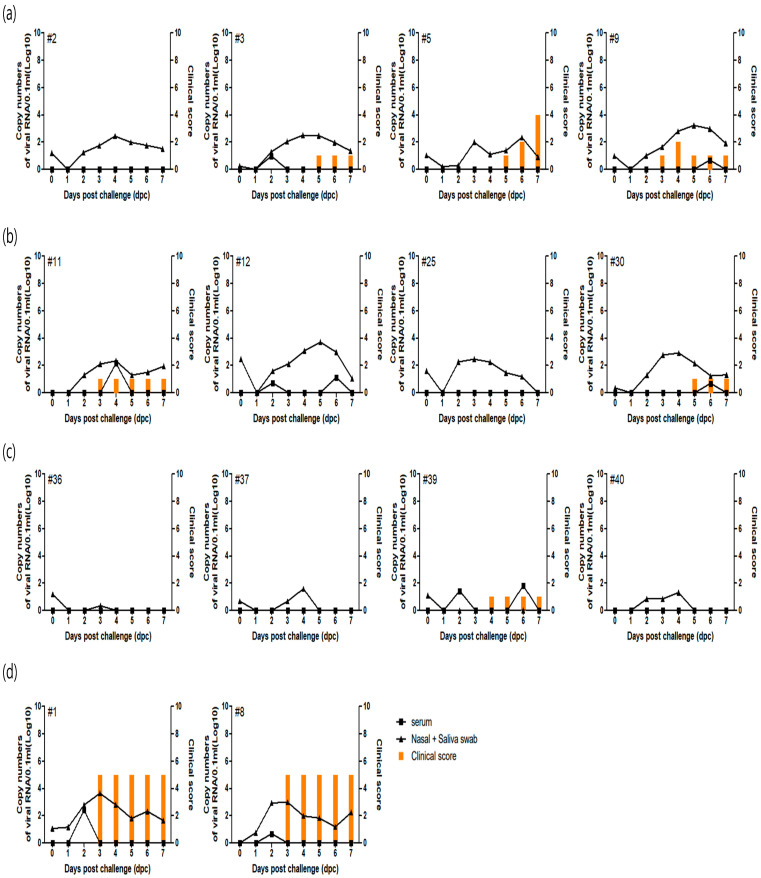
Evaluation of clinical signs and viral shedding in pigs vaccinated with commercial FMD vaccine following O/HKN/5/2019 challenge. (**a**) G1: O1/Manisa + O/3039 vaccinated, (**b**) G2: O/Primorsky vaccinated, (**c**) G3: O1/Campos vaccinated, (**d**) G4: unvaccinated. Each graph shows the pig identification number in the upper-left corner. Serum samples were collected every other day, and combined nasal and saliva swab samples were obtained daily starting on the day of challenge.

Among the groups, one of four pigs in G1 (O1/Manisa + O/3039), two of four in G2 (O/Primorsky), and three of four in G3 (O1/Campos) were fully protected and exhibited no clinical signs, whereas no pigs in the unvaccinated group (G4) were protected against the challenge. All animals in the vaccinated groups that developed clinical signs had very low clinical scores (≤2), except for one pig (G1 #5). Additionally, the unvaccinated group exhibited severe clinical signs starting at 3 days post-challenge (dpc), whereas clinical signs in the vaccinated groups appeared later, at 4 or 5 dpc. Overall, vaccinated groups exhibited lower clinical scores and a delayed onset of clinical signs compared with the unvaccinated group (Figure 1).

Virus shedding was detected in all pigs based on combined nasal and saliva swab samples. In G3, the viral RNA copy number (log_10_) ranged from 0.34 to 1.6, whereas in the unvaccinated group (G4), values ranged from 1.75 to 3.7. Transient viremia was observed in G1 (#3, #9), G2 (#11, #12, #30), G3 (#39), and in all pigs from the unvaccinated group (G4). The viral RNA copy numbers (log_10_) in vaccinated groups ranged from 0.36 to 2.16, while those in the unvaccinated group ranged from 0.69 to 2.42 (Figure 1).

### 3.2. ELISA-Based Immunogenicity Assessment

**Figure 2:**
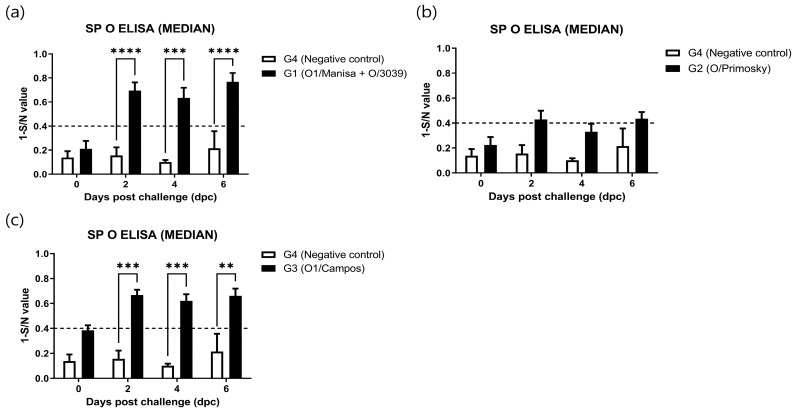
Structural protein (SP) antibody levels after challenge with the O/HKN/5/2019 virus compared between vaccinated and unvaccinated groups. (**a**) G1 (O1/Manisa + O/3039) vs. G4 (negative control, unvaccinated), (**b**) G2 (O/Primorsky) vs. G4, (**c**) G3 (O1/Campos) vs. G4. The dashed line indicates the threshold for seropositivity, and values above the dashed line are considered positive. Data were subjected to mixed-effects analysis, followed by Tukey’s post hoc test for multiple comparisons. ** *p* < 0.01, *** *p* < 0.001 and **** *p* < 0.0001.

The unvaccinated group (G4; negative control) tested negative for type O SP antibodies. Although all vaccinated groups were also negative at the time of challenge, G3 exhibited the highest antibody values, approaching the threshold for seropositivity. By 2 dpc, all vaccinated groups had seroconverted; however, the antibody levels in G2, although positive, remained relatively lower than those observed in the other vaccinated groups (Figure 2).

### 3.3. Serological Response Assessed by VNT

**Figure 3:**
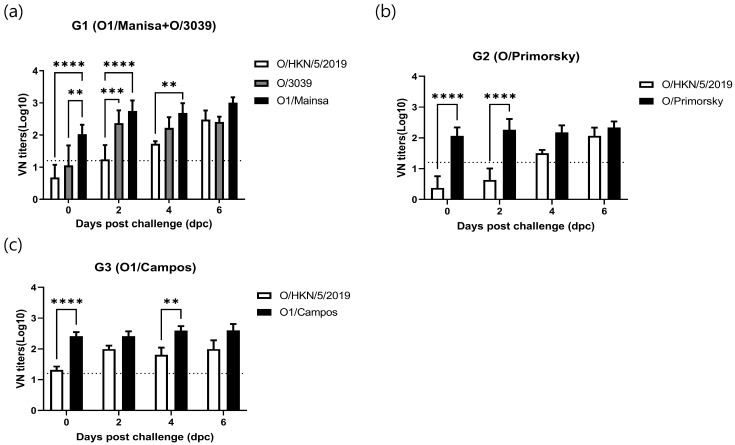
Virus neutralization test (VNT) results obtained using the challenge virus and vaccine strain viruses. VN titers are shown for each vaccination group. (**a**) O/3039 vs. O1/Manisa vs. O/HKN/5/2019, (**b**) O/Primorsky vs. O/HKN/5/2019, (**c**) O1/Campos vs. O/HKN/5/2019. The dashed line indicates the threshold for seropositivity, and values above the dashed line are considered positive. Data were subjected to two-way repeated measures analysis of variance (ANOVA), followed by Tukey’s post hoc test for multiple comparisons. ** *p* < 0.01; *** *p* < 0.001 and **** *p* < 0.0001.

VN titers were measured against the challenge virus and each vaccine strain virus, and compared within each vaccinated group. Analysis of VN titers (log_10_) against the challenge virus (O/HKN/5/2019) revealed that only G3 seroconverted at the time of challenge, with a titer of 1.32. G1 and G2 seroconverted at 2 and 4 dpc, respectively, showing titers of 1.24 and 1.5, which were above positive levels (Figure 3 and Table S1).

VN titers against each vaccine strain virus, except O/3039, showed seroconversion from 0 dpc, with titers at or above positive levels. In G1, comparison of VN titers against O/HKN/5/2019 and the vaccine strains O1/Manisa and O/3039 revealed the highest titer against O1/Manisa. In G2 and G3, O/Primorsky and O1/Campos, respectively, showed higher VN titers than O/HKN/5/2019. The VN titers were 2.03 for O1/Manisa, 2.07 for O/Primorsky, and 2.41 for O1/Campos (Figure 3).

## 4. Discussion

Among the FMDV serotypes circulating in Pool 1, serotype O accounts for more than 80% of reported outbreaks, with the O/ME-SA (O/ME-SA/Ind-2001 and O/ME-SA/PanAsia), O/SEA (O/SEA/Mya-98), and O/CATHAY topotypes being most frequently detected [28,29]. Among these, the O/CATHAY topotype is recognized as porcinophilic. Analyses of host species distribution over time have shown that approximately 97.9% of O/CATHAY detections occur in pigs, whereas only 2.1% are reported in cattle [21,30]. In addition, O/CATHAY has been detected in the PR China and several Southeast Asian countries and is reported to evolve at a higher rate than other topotypes, resulting in increased genetic diversity [21]. Between 2013 and 2020, O/CATHAY was identified in neighboring regions, including Vietnam (2016–2018), Hong Kong (2013–2019), and PR China (2013, 2016, and 2018) [31,32]. Furthermore, the 2022 annual report of the WOAH–Food and Agriculture Organization FMD Reference Laboratory Network indicated continued detection of O/CATHAY in PR China from 2020 to 2022 [33]. Owing to the geographic proximity of these regions to the ROK, these findings suggest an increased likelihood of O/CATHAY introduction into the ROK. Therefore, it is essential to evaluate whether the commercial vaccines currently used in the ROK provide sufficient protection against this topotype. In this context, the present study assessed the protective efficacy of three commercially available FMD vaccines used in the ROK against the O/CATHAY topotype.

FMDV can be detected in tissues and secretions even in the absence of overt clinical lesions [8]. In particular, pigs act as major amplifiers of FMDV, releasing up to 3000 times more aerosolized virus than cattle [34]. In the present study, pigs were housed in groups without isolating individuals that developed clinical signs. Under these conditions, secondary exposure to aerosolized virus shed by infected animals may have occurred, particularly in pigs showing delayed onset of clinical signs. Despite these conditions, all three vaccines demonstrated protective efficacy against the O/CATHAY topotype. Among them, the O1/Campos vaccine (G3) showed the highest level of protection, as evidenced by the fewest clinical signs and a significant reduction in viral shedding compared with the unvaccinated group. Consistently, ELISA and VNT results showed that this group had the highest antibody titers at the time of challenge.

Although the O1/Campos vaccine demonstrated the highest protective efficacy, it did not confer complete protection. These findings suggest that this vaccine may be suitable for emergency use; however, the development of vaccines specifically targeting the O/CATHAY topotype remains necessary. Given the limited sample size of this study, further investigations are required to substantiate these findings, including studies involving larger animal cohorts or evaluations conducted under field conditions. In addition, continuous monitoring of the emergence and evolution of FMDV topotypes in Pool 1 is essential for effective FMD control and preparedness, enabling timely responses to emerging risks.

## 5. Conclusions

This study demonstrates that the three commercial FMD vaccines evaluated provided only partial protection against the O/CATHAY topotype. Among them, the O1/Campos vaccine exhibited the strongest protective effect, as indicated by the fewest clinical signs, significantly reduced viral shedding, and higher antibody titers compared with the other vaccines. However, because full protection was not achieved, this vaccine may be considered an emergency measure rather than a definitive solution. Consequently, further research aimed at developing vaccines specifically targeting the O/CATHAY topotype is warranted.

## Figures and Tables

**Table 1:** Experimental design to assess the effectiveness of three commercial FMD vaccines.

Groups	No. of Animals.	Vaccine Strains	Injected Volume (mL)	Day of Vaccination	Serum Obtained at dpc	Swab Obtained at dpc
G1	4	O1/Manisa + O/3039 + A22/Iraq	2	−21 (prime), −7 (boost)	−21, −14, −7, 0, 2, 4, 6	0, 1, 2, 3, 4, 5, 6, 7
G2	4	O/Primorsky + A/Zabaikalsky	2	−21 (prime), −7 (boost)	−21, −14, −7, 0, 2, 4, 6	0, 1, 2, 3, 4, 5, 6, 7
G3	4	O1/Campos + A24/Cruzeiro + A/Argentina	2	−21 (prime), −7 (boost)	−21, −14, −7, 0, 2, 4, 6	0, 1, 2, 3, 4, 5, 6, 7
G4	2	Unvaccinated	-	-	−21, −14, −7, 0, 2, 4, 6	0, 1, 2, 3, 4, 5, 6, 7

dpc, days post-challenge; prime, day of prime vaccination; boost, day of boost vaccination.

## Data Availability

The original contributions presented in this study are included in the article/Supplementary Materials. Further inquiries can be directed to the corresponding authors.

## Supplementary Materials

The following supporting information can be downloaded at: https://www.mdpi.com/article/10.3390/microorganisms14010186/s1, Table S1: Summary of clinical signs and laboratory tests in vaccinated and challenged pigs.
