# Supplementary material for: Evaluation of the Protective Efficacy of Foot-and-Mouth Disease Vaccines Against O/CATHAY Topotype Virus in Pigs
Source: Microorganisms. 2026 Jan 14;14(1):186. doi: 10.3390/microorganisms14010186 (PMC12844367; doi:10.3390/microorganisms14010186)
Supplement: Supplementary file 1 [file microorganisms-14-00186-s001.zip › microorganisms-3975212-supplementary.pdf]

Table S1. Summary of clinical signs and laboratory tests in vaccinated and challenged pigs

Groups	Pig ID	VN titer against O/HKN/5/2019 at 0 dpc *	Clinical score †	Virus detection by rRT-PCR for 7 days after challenge	
				Nasal swabs	Sera
G1	#2	0	0	+	-
	#3	0	1	+	+
	#5	1.20	4	+	-
	#9	1.51	2	+	+
G2	#11	0	1	+	+
	#12	0	0	+	+
	#25	0	0	+	-
	#30	1.51	1	+	+
G3	#36	1.20	0	+	-
	#37	1.20	0	+	-
	#39	1.20	1	+	+
	#40	1.65	0	+	-
C4	#1	0	5	+	+
	#8	0	5	+	+

VN, virus neutralizing; rRT-PCR, real-time reverse transcription-polymerase chain reaction; dpc, days post-challenge.
* challenged with O/HKN/5/2019 at 0 dpc; † Clinical scores were based on the sum of each FMD lesion or sign (maximum score = 8) according to the method reported by Kim et al. (2023) based on the addition of point.
